# Resilient Identity: How Aging Matters in Exploring Resilience

**DOI:** 10.1093/geroni/igaa057.3045

**Published:** 2020-12-16

**Authors:** Lauren Bouchard, Lydia Manning

**Affiliations:** 1 Concordia University Chicago, Chicago, Illinois, United States; 2 Concordia University Chicago, River Forest, Illinois, United States

## Abstract

Defining resilience is complex given its multidimensional and contextualized nature within the gerontological literature. The construct has been described as a trait, state, and process, and less often, as a cultivated identity. Older adults are key in the understanding of resilience from their own point view as experts of their experiences with adversity. This presentation focuses upon the findings of qualitative research utilizing grounded theory methodology, which explores the way aging may shape “resilient identity.” Given the varying challenges across the life course, resilient identity may be highly dependent on individual and environmental context. This talk also focuses on who determines the definition of resilience as well as the nature of such an identity. Educational implications regarding key factors, management strategies, and protective practices are also discussed.

